# Construction of an Immune-Related lncRNA Signature That Predicts Prognosis and Immune Microenvironment in Osteosarcoma Patients

**DOI:** 10.3389/fonc.2022.769202

**Published:** 2022-04-14

**Authors:** Yi He, Haiting Zhou, Haoran Xu, Hongbo You, Hao Cheng

**Affiliations:** ^1^ Department of Orthopedics, Tongji Hospital, Tongji Medical College, Huazhong University of Science and Technology, Wuhan, China; ^2^ Department of Oncology, Tongji Hospital, Tongji Medical College, Huazhong University of Science and Technology, Wuhan, China

**Keywords:** immune, long non-coding RNA, osteosarcoma, TCGA, signature

## Abstract

Osteosarcoma is one of the most common bone tumors in teenagers. We hope to provide a reliable method to predict the prognosis of osteosarcoma and find potential targets for early diagnosis and precise treatment. To address this issue, we performed a detailed bioinformatics analysis based on the Cancer Genome Atlas (TCGA). A total of 85 osteosarcoma patients with gene expression data and clinicopathological features were included in this study, which was considered the entire set. They were randomly divided into a train set and a test set. We identified six lncRNAs (ELFN1-AS1, LINC00837, OLMALINC, AL669970.3, AC005332.4 and AC023157.3), and constructed a signature that exhibited good predictive ability of patient survival and metastasis. What’s more, we found that risk score calculated by the signature was positively correlated to tumor purity, CD4^+^ naive T cells, and negatively correlated to CD8^+^ T cells. Furthermore, we investigated each lncRNA in the signature and found that these six lncRNAs were associated with tumorigenesis and immune cells in the tumor microenvironment. In conclusion, we constructed and validated a signature, which had good performance in the prediction of survival, metastasis and immune microenvironment. Our study indicated possible mechanisms of these lncRNAs in the development of osteosarcoma, which may provide new insights into the precise treatment of osteosarcoma.

## Introduction

Osteosarcoma is one of the most common bone tumors, most commonly occurring in young children and adolescents (1). This tumor is most likely to happen in the metaphyses of the distal femur, proximal tibia, and proximal humerus (2–4). Osteosarcoma is prone to pulmonary metastases, and 20% of patients are found to have pulmonary metastases at the time of initial diagnosis (5). Medical advances have significantly reduced the mortality rate of osteosarcoma patients. However, the lack of specific markers makes early screening for osteosarcoma still difficult. Treatment of osteosarcoma is mainly based on local excision and chemotherapy, but chemotherapy for osteosarcoma is prone to drug resistance and has great toxic side effects (6–8). As a result, overall survival is still not satisfactory. It is urgent to figure out the mechanism of tumorigenesis, metastasis and drug resistance in osteosarcoma, and to discover potential target for earlier diagnosis and gene therapy.

The immune system is an important part of the human body. It can help us fight against pathogen infections and participate in the monitoring and prevention of cancer, playing an essential role in maintaining the integrity of the body. However, some tumor cells can evade the surveillance by immune system, or suppress the immune response, making cancer progression. Meanwhile, more and more evidence has shown that the imbalance of immune state in tumor microenvironment plays a decisive role in tumor development (9). Therefore, the role of immune-related factors in tumor development deserves to be studied.

LncRNAs are highly heterogeneous RNA characterized by their length of more than 200 nucleotides and do not encode proteins. With advances in gene chip technology, lncRNAs are being rapidly identified. Numerous studies have found that lncRNAs are involved in various physiological processes, including cellular differentiation, immune response, and tumor progression (10, 11). LncRNAs appear to play an important role in tumor progression, exhibiting tumor-inhabiting and tumor-promoting functions (12). LncRNA-ATB was reported to functions as a tumor promoter in papillary thyroid cancer (13). LOC285194 was reported to function as a tumor suppressor in non-small cell lung cancer and osteosarcoma (14, 15). As a result, lncRNAs are expected to be new biomarkers and therapeutic targets for cancer (16).

**Figure 1:**
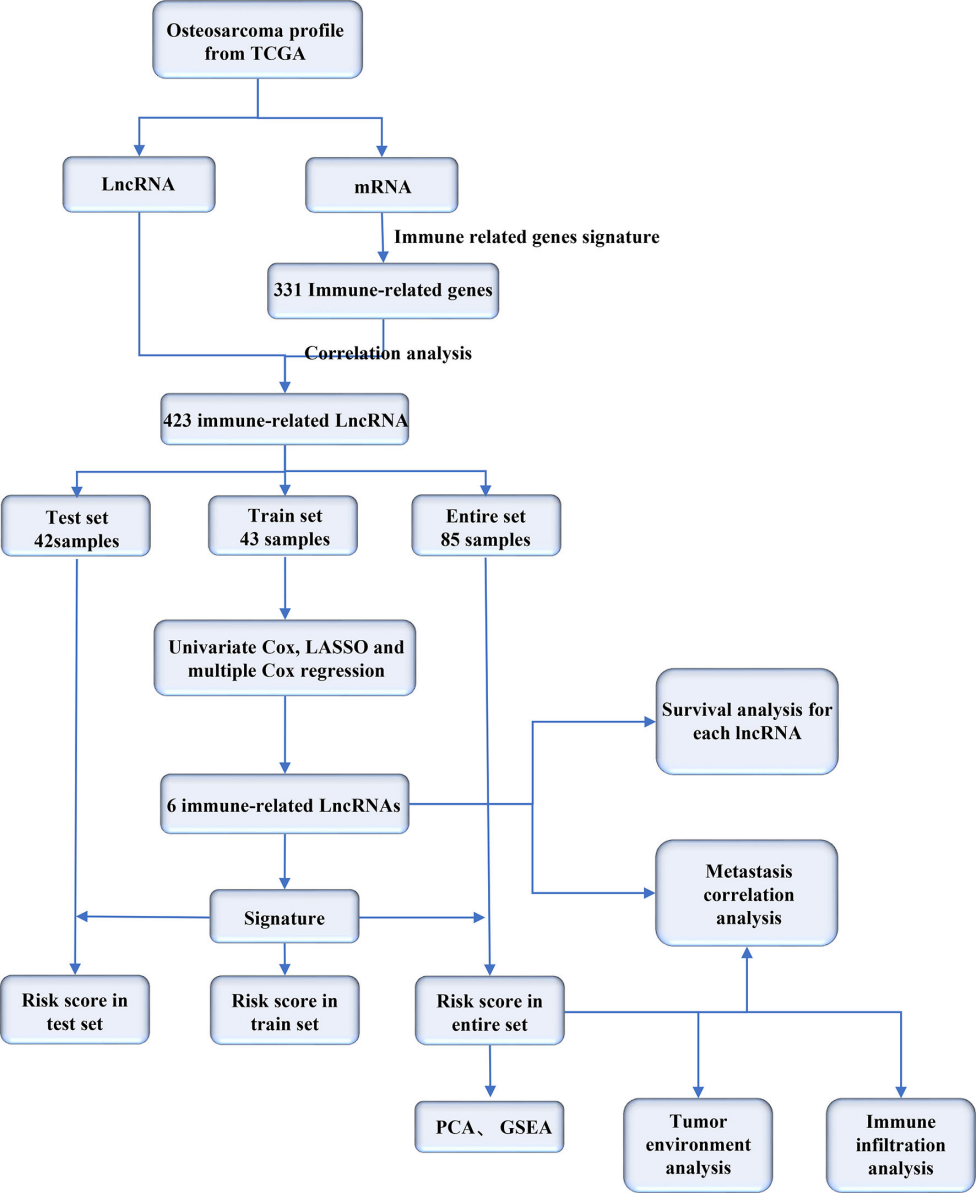
Flowchart of our study.

There was little research studied on the immune-related lncRNAs in osteosarcoma. In this study, we screened immune-related lncRNAs in osteosarcoma and made a bioinformatics analysis for them on the basis of TCGA data. The flowchart of our study is as follows (**Figure 1**).

## Materials and Methods

### Sample Datasets

The RNAseq (level 3) data and corresponding clinical data of osteosarcoma were come from the TCGA database (https://portal.gdc.cancer.gov/), and all data was normalized by TMM method. We used the Ensembl Genome Browser website to annotate the mRNAs and lncRNAs in the expression matrix and subsequently extracted the mRNA and lncRNA expression matrix separately. To make the analysis accurate, we removed the samples with incomplete clinical information and overall survival time lower than 30 days (17). In total, 85 cases were contained for the following study. All TCGA data is available to the public, so there is no further approval needed from the Ethics Committee.

### Immune-related lncRNA Acquisition

We acquired immune-related genes from the Molecular Signature Database v 7.1 (MSigDB) (http://www.broad.mit.edu/gsea/msigdb/) (17, 18). IMMUNE_RESPONSE.gmt and IMMUNE_SYSTEM_PROCESS.gmt were chosen as the annotated gene sets. We removed overlapped genes and ultimately obtained 331 immune-related genes. The expression information of these 331 immune-related genes was extracted from the mRNA expression matrix. Then Pearson correlation analysis was performed to calculate the correlation coefficient of each lncRNA with immune-related mRNAs. LncRNAs with coefficient > 0.6 and P value < 0.001 were defined as immune-related lncRNAs. Finally, the expression matrix of these immune-related lncRNAs was extracted.

### Signature Construction

These 85 cases were separated into a train set and a test set randomly by “caret” package of R software (v 3.6.2). Meanwhile, all 85 cases were taken as the entire set. The train set was used to construct the prognostic signature. The test set and the entire set were used to verify the accuracy of the signature.

In the train set, we performed univariate Cox regression analysis to identify prognostic-related lncRNAs with p<0.01. Then Least absolute shrinkage and selection operator (LASSO) regression was performed to remove lncRNAs that could lead to phenomenon of overfitting. Finally, the lncRNAs was further screened by using multivariate Cox regression analysis. The coefficient, HR value and P value for each lncRNA were calculated. The risk score of each sample was calculated by the signature:


Risk score = β(gene1) ∗ expr(gene1) + β(gene2)∗  expr(gene2) +···+ β(genen) ∗ expr(genen)

β is the coefficient of lncRNA, and expr(genen) stands for the expression value of lncRNA.

### Signature Application and Validation

Firstly, we did the analysis in the train set. We used the formula above to calculate the risk score of each patient and divided patients into high or low risk groups according to the risk score by using “survminer” package. We ranked the patients by the risk score and plotted the dot-plot for the survival status of each patient. We mapped the lncRNA expression heatmap to observe the expression of lncRNAs in the high and low risk groups. We applied Kaplan-Meier method to explore the survival differences between high-risk group and low-risk group. Finally, receiver operating characteristic (ROC) curve of 5-years was applied for identifying the diagnostic value of the risk scoring signature.

Subsequently, the same analysis was performed in the test set and the entire set to verify the accuracy of the signature.

### Comprehensive Analysis in the Entire Set

The entire set was used for the subsequent analysis. Firstly, principal component analysis (PCA) was used to test whether the signature can better distinguish the risk status. Secondly, survival analysis for each lncRNA in the signature was performed to estimate the effect of individual lncRNA on survival. Thirdly, metastasis correlation analysis was carried out to investigate the potential correlation between risk score and metastasis. Fourthly, Gene set enrichment analysis (GSEA; https://www.gsea-msigdb.org/gsea/index.jsp) (17) was performed based on two gene sets (IMMUNE_RESPONSE.gmt and IMMUNE_SYSTEM_PROCESS.gmt) to analyze immune response enrichment in the two groups. Fifthly, ESTIMATE algorithm (19) was used to assess the proportion of immune and stromal components, and to infer the tumor purity of the samples. We subsequently analyzed the relationship between tumor purity and risk scores. Finally, CIBERSORT method (20) was used to analyze the infiltration of 22 immune cells between high and low risk groups. The correlation between the infiltrating proportion of immune cells and risk scores and the lncRNAs were also analyzed.

All analyses were performed on the R software (version 3.6.2, https://www.r-project.org/). And P < 0.05 was considered statistically significant.

## Results

### Construction of Signature

**Figure 2:**
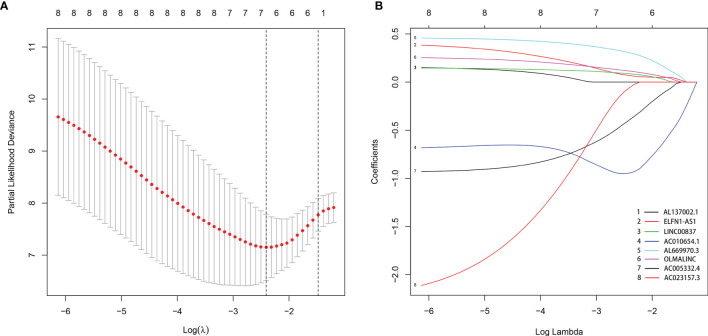
The LASSO regression analysis. When -3 < lambda < -2, the partial likelihood deviation was smallest **(A)**, at which point AL137002.1 was excluded **(B)**.

By comparing mRNA expression data with two gene sets from MSigDB, we matched 331 immune-related genes. We calculated the expression correlation between lncRNAs and immune-related genes to obtain the immune-related lncRNAs. Subsequently, 423 immune-related lncRNAs was identified. Eight of them were screened out by univariate Cox regression analysis (**Table 1**). The result of the LASSO regression analysis showed that the partial likelihood deviation was smallest when -3 < lambda < -2 (**Figure 2A**), at which point AL137002.1 was excluded (**Figure 2B**). Finally, six lncRNAs were screened from the remaining seven lncRNAs by using multivariate Cox regression analysis, and their coefficients, HR values and p-values were calculated respectively (**Table 2**). The signature was constructed with the coefficients of lncRNAs as below.


Risk score=(0.4707∗ELFN1−AS1)+(0.1634∗LINC00837)+(0.4341∗AL669970.3)+(0.2696∗OLMALINC)+(−1.1261∗AC005332.4)+(−2.2230∗AC023157.3).

**Table 1:** Eight lncRNAs obtained after univariable Cox regression analysis.

LncRNA	HR	HR.95L	HR.95H	P value
AL137002.1	1.9475	1.1789	3.2173	0.0093
ELFN1-AS1	1.3253	1.1129	1.5783	0.0016
LINC00837	1.1374	1.0336	1.2516	0.0084
AC010654.1	0.0796	0.0139	0.4565	0.0045
AL669970.3	1.6130	1.1893	2.1877	0.0021
OLMALINC	1.3049	1.0831	1.5721	0.0051
AC005332.4	0.2580	0.0965	0.6900	0.0070
AC023157.3	0.2577	0.1002	0.6630	0.0049

HR: Hazard ratio.

HR.95L: Lower 95% confidence interval of hazard ratio.

HR.95H: Higher 95% confidence interval of hazard ratio.

**Table 2:** Six lncRNAs obtained after multivariable Cox regression analysis.

LncRNA	Coef	HR	HR.95L	HR.95H	P value
ELFN1-AS1	0.4704	1.6006	1.1239	2.2794	0.0091
LINC00837	0.1634	1.1775	1.0381	1.3357	0.0110
AL669970.3	0.4341	1.5436	1.0049	2.3708	0.0474
OLMALINC	0.2696	1.3094	1.0390	1.6502	0.0223
AC005332.4	-1.1261	0.3243	0.0771	1.3640	0.1244
AC023157.3	-2.2230	0.1083	0.0215	0.5447	0.0070

Coef: Coefficient.

HR: Hazard ratio.

HR.95L: Lower 95% confidence interval of hazard ratio.

HR.95H: Higher 95% confidence interval of hazard ratio.

Among these lncRNAs, five immune-related lncRNAs were independent prognostic factors. One immune-related lncRNAs acted as a complement to others.

### Validation of the Signature

**Figure 3:**
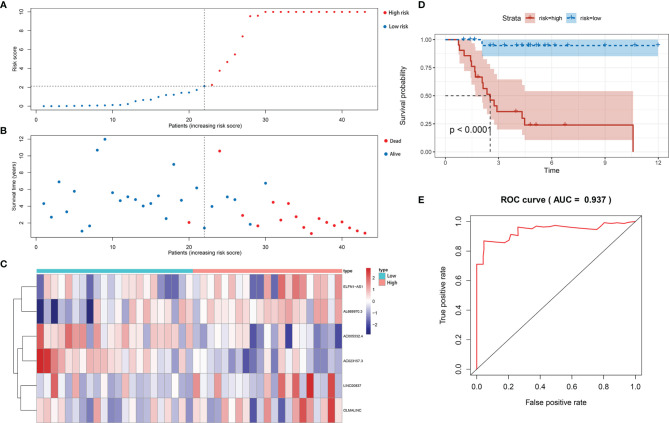
Construction of immune-related lncRNA signature for osteosarcoma. **(A)** The risk curve of each patient reordered by risk score in train set. **(B)** The scatter plot of all patient’s survival state in train set. **(C)** The heatmap showed the expression levels of six lncRNAs between the low-risk group and high-risk group in train set. **(D)** Patients in the high-risk group indicated worse overall survival than those in the low-risk group in train set. **(E)** AUC for risk score of 5-year survival according to the ROC curves in train set.

We calculated the risk score of each sample by the signature and divided patients into the low-risk and the high-risk groups by the median value of risk score. We ranked patient by the risk score (**Figure 3A**) and plotted the dot-plot for the survival status of each patient (**Figure 3B**). The results showed that the higher risk score patients had, the shorter survival time patients might have. According to the heatmap, the expression levels of ELFN1-AS1, LINC00837, OLMALINC and AL669970.3 were higher in the high-risk group, while AC005332.4 and AC023157.3 were higher in the low-risk group (**Figure 3C**). The survival curve shows that the patients in the high-risk group had a lower survival time (**Figure 3D**). The area under curve (AUC) of 5 years in the train set was 0.937 (**Figure 3E**).

**Figure 4:**
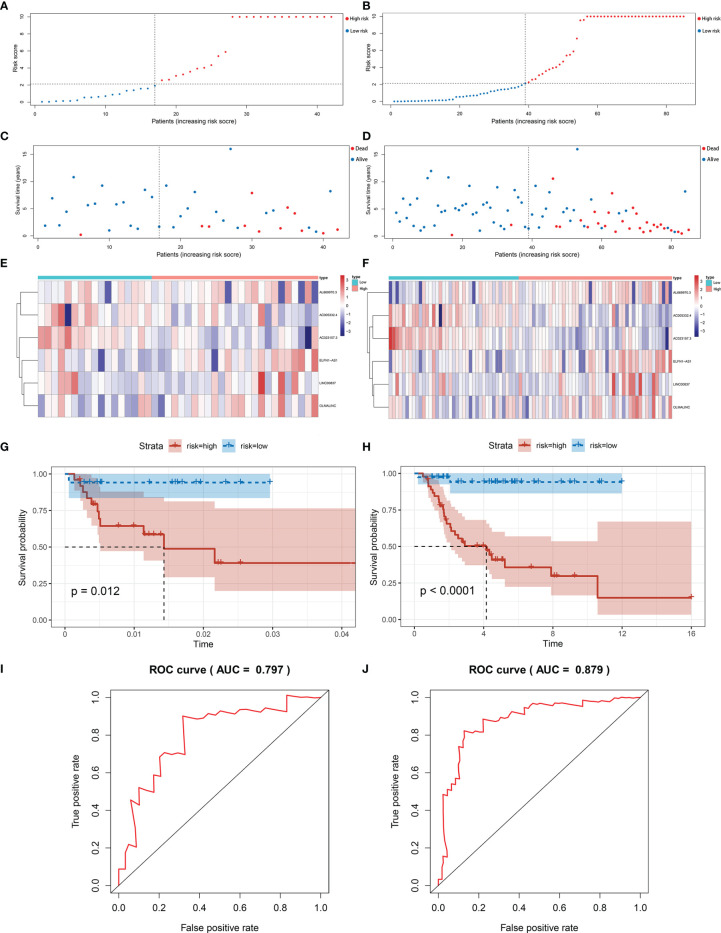
Validation of immune-related lncRNA signature for osteosarcoma. **(A, B)** The risk curve of each patient reordered by risk score in test set and entire set. **(C, D)** The scatter plot of all patient’s survival state in test set and entire set. **(E, F)** Patients in the high-risk group indicated worse overall survival than those in the low-risk group in test set and entire set. **(G, H)** The heatmap showed the expression levels of six lncRNAs between the low-risk group and high-risk group in test set and entire set. **(I, J)** AUC for risk score of 5-year survival according to the ROC curves in test set and entire set.

The same signature was used in the test set and the entire set. And we got similar results in the two sets as we expected. Patients in the low-risk group have better overall survival. The results in the test set (**Figures 4A, C, E, G**) and the entire set (**Figures 4B, D, F, H**) were presented in the figure below. The area under curve (AUC) of 5 years in the test set was 0.797, and 0.879 in the entire set (**Figures 4I, J**).

**Figure 5:**
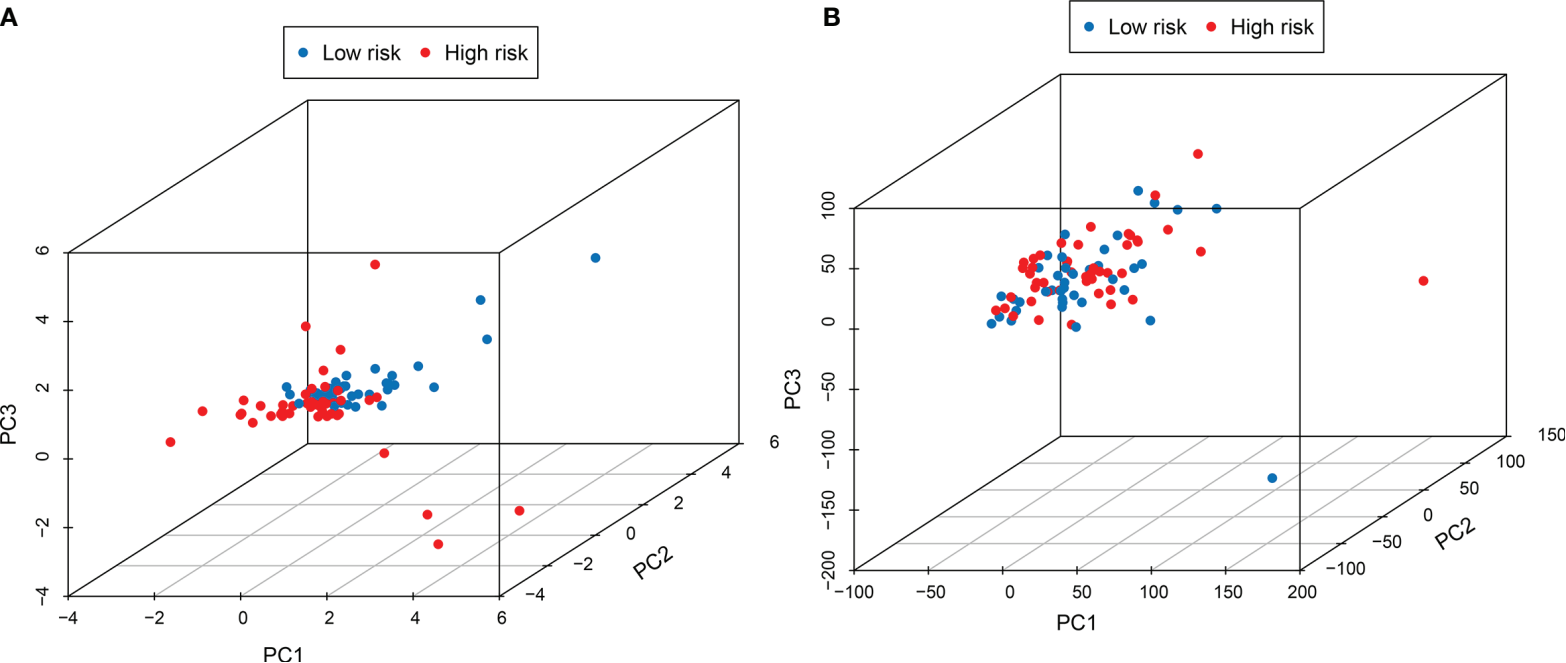
PCA analysis showed that the use of this signature **(A)** could better distinguish the risk status of patients than the use of the whole genome **(B)**.

In order to test whether the signature can better distinguish the risk status, PCA analysis was carried out using the signature and genome-wide expression. When using the signature, the risk status of the patients was separated well (**Figure 5A**). While it did not display a clear separation when using the whole genome expression (**Figure 5B**).

### Survival Analysis for lncRNAs in the Signature

**Figure 6:**
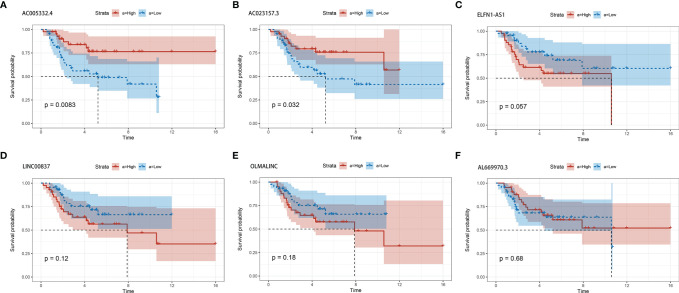
The survival curves for each of the six lncRNAs. **(A)** AC005332.4. **(B)** AC023157.3. **(C)** ELFN1-AS1. **(D)** LINC00837. **(E)** OLMALINC. **(F)** AL669970.3.

In order to estimate the effect of expression of individual lncRNAs in the signature, we performed survival analysis for each lncRNA in the signature. Patients with higher expression of AC005332.4 and AC023157.3 indicated better overall survival than those with lower expression (**Figures 6A, B**). However, the results for other lncRNAs were not statistically significant (**Figures 6C–F**).

### Metastasis Correlation Analysis

**Figure 7:**
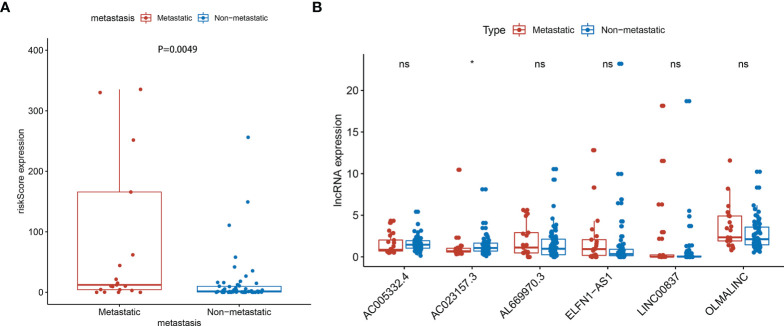
Metastasis correlation analysis in the entire sets. **(A)** The risk score was correlated with metastatic (P=0.0049) (Some outliers are not shown in the figure). **(B)** AC023157.3 was highly correlated with metastasis (P<0.05). *: P<0.05, ns: P>0.05.

The prognosis of osteosarcoma is strongly related to the presence of metastasis, so it is important to verify whether the signature is predictive of metastasis. We carried out metastasis correlation analysis to investigate the potential correlation between risk score and metastasis in the entire set. The result showed that the risk score was correlated to metastatic status (P=0.0049) (**Figure 7A**). And one of the lncRNA, AC023157.3, was down-regulated in metastatic patients (P<0.05) (**Figure 7B**).

### Analysis of Tumor Environment and Immune Infiltration

**Figure 8:**
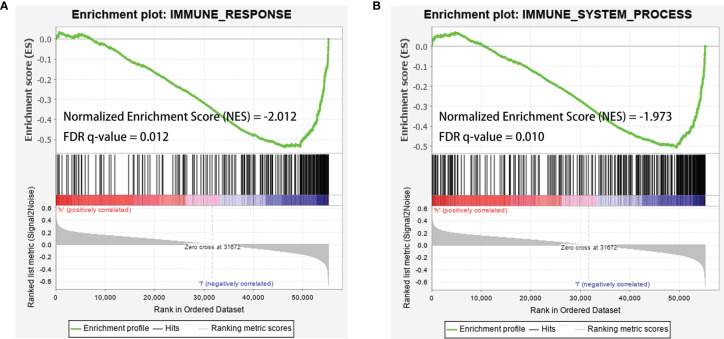
GSEA analysis showed that immune-related responses were enriched in the low-risk groups. **(A)** GSEA analysis based on IMMUNE_RESPONSE gene set. **(B)** GSEA analysis based on IMMUNE_SYSTEM_PROCESS gene set. The green line was the running enrichment score (ES) for the gene set as the analysis walks down the ranked list. A positive value of ES indicates that the gene set was enriched in the high risk group, and a negative value of ES indicates that the gene set was enriched in the low risk group. The ranking metric score indicated a gene’s correlation with a phenotype. A positive value refers to high risk and a negative value refers to low risk. NES, normalized enrichment score; FDR, false discovery rates.

**Figure 9:**
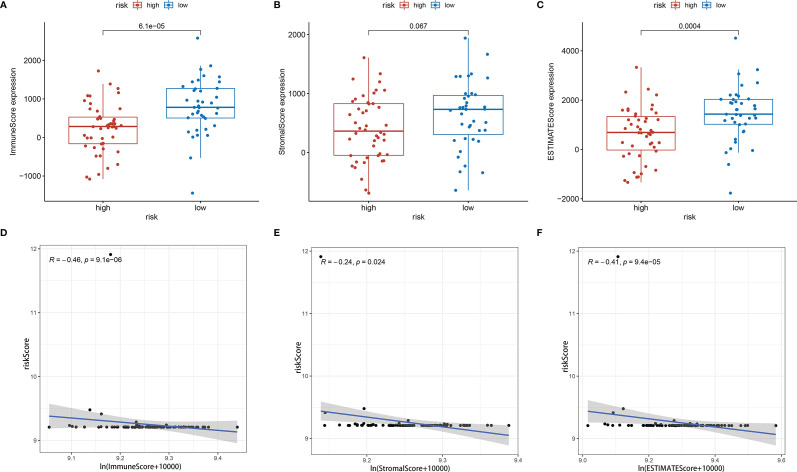
Tumor microenvironment analysis showed risk score was negatively correlated with tumor purity. **(A)** Immune score between high-risk group and low-risk group. **(B)** Stromal score between high-risk group and low-risk group. **(C)** ESTIMATE score between high-risk group and low-risk group. **(D–F)** Immune score, stromal score and ESTIMATE score were negatively correlated with risk score.

**Figure 10:**
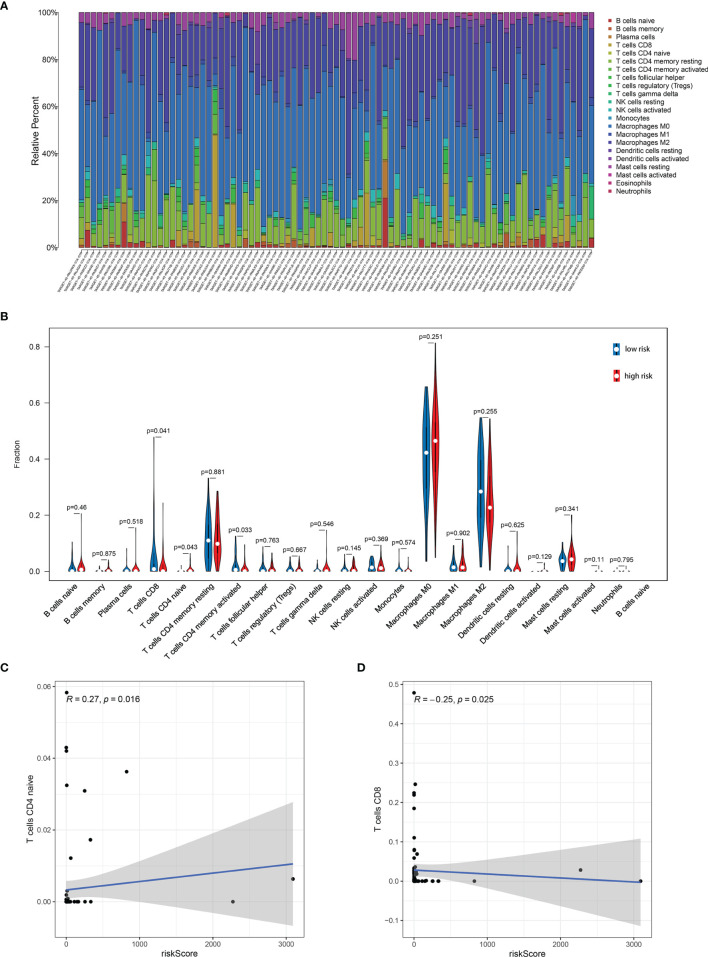
Immune infiltration analysis showed the relationship between risk score and immune cells. **(A)** The main components of the immune environment were T-lymphocytes and macrophages in osteosarcoma. **(B)** The composition of immune cells between high-risk group and low-risk group. **(C, D)** Risk score was negatively correlated to CD8^+^ T cells and positively correlated to CD4^+^ naive T cells.

**Figure 11:**
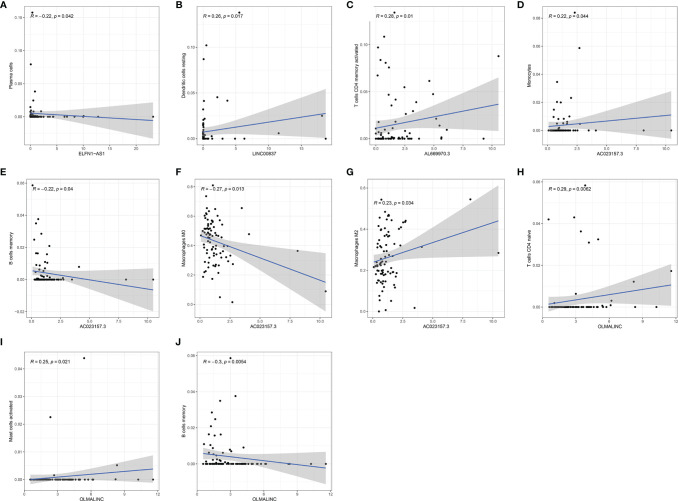
The relationship of each gene in the signature to the immune cells. **(A)** ELFN1-AS1. **(B)** LINC00837. **(C)** AL669970.3. **(D–G)** AC023157.3. **(H–J)** OLMALINC.

Further analysis by GSEA showed that immune response pathways were enriched in the low-risk groups (**Figures 8A, B**). The immune score calculated by ESTIMATE algorithm suggested that immune component was significantly higher in the low-risk group (P<0.01) (**Figure 9A**). The stromal score was also higher in the low-risk group, but it did not achieve statistical significance (P>0.05) (**Figure 9B**). The ESTIMATE score was higher in the low-risk group, suggesting the lower tumor purity in low-risk group (**Figure 9C**). We also found that the risk score was negatively correlated with immune score, stromal score and ESTIMATE score by correlation analysis (**Figures 9D–F**). We calculated the proportion of 22 immune cell in each sample using the CIBERSORT algorithm. The results showed the main components of the immune environment were T lymphocytes and macrophages (**Figure 10A**). We then group the samples according to the risk scores, and found that the high-risk group was enriched with CD4^+^ naive T cells (P=0.043), while the low-risk group was enriched with CD8^+^ T cells (p=0.041) and CD4^+^ activated memory T cells (p=0.033) (**Figure 10B**). In addition, we also analyzed the correlation between risk scores and 22 immune cells, and found risk scores was negatively correlated to CD8^+^ T cells and positively correlated to CD4^+^ naive T cells (**Figures 10C, D**). We then also analyzed the relationship between each gene in the signature and the proportion of immune cells. ELFN1-AS1 was negatively related to plasma cells (**Figure 11A**). LINC00837 was positively related to resting dendritic cells (**Figure 11B**). AL669970.3 was positively related to activated CD4^+^ memory T cells (**Figure 11C**). AC023157.3 was positively related to monocytes and M2 macrophages, and negatively related to memory B cells and M0 macrophages (**Figures 11D–G**). OLMALINC was positively related to activated mast cells, CD4^+^ naive T cells and negatively related to memory B cells (**Figures 10H–J**). AC005332.4 did not show association with immune cells.

## Discussion

Although the application of new treatment has increased overall survival in osteosarcoma, the prognosis for patients, especially the metastatic and recurrent patients, remains poor due to the lack of specific biomarkers and therapeutic target for early diagnosis and precise treatment. Therefore, it is urgent to find specific biomarkers and therapeutic target for osteosarcoma. Studies have emphasized that the immune response in the microenvironment plays a crucial role in the development of a variety of cancers, and lncRNAs are an important regulator of the immune response (21, 22). Many studies have also reported that lncRNAs were involved in the proliferation, metastasis and drug resistance of osteosarcoma (14, 23–26). There are also emerging evidence indicating that immune-related lncRNAs are valuable in predicting prognosis and also maybe targets for specific treatment (27, 28).

Based on a TCGA dataset, we included 85 samples with complete clinical information in our study. Then we identified a potential prognostic six-lncRNAs signature. This signature included ELFN1-AS1, LINC00837, OLMALINC, AL669970.3, AC005332.4 and AC023157.3. It was of great value in predicting the prognosis of patients in all set as we hope. The 5-years AUC of ROC was 0.879 in the entire set. The prognosis of almost all tumors is highly correlated with metastasis, especially osteosarcoma. We found that the risk score based on the signature was highly correlated with metastasis, suggesting that signature is also a better predictor of osteosarcoma metastasis. It might be one of the reasons that this signature is such a good predictor of patient outcomes. Principal component analysis showed that the patient’s risk status can be well differentiated when using this signature. Moreover, we found that AC005332.4 and AC023157.3 were mainly expressed in the low-risk groups, and they may improve the overall survival of patient. While ELFN1-AS1, LINC00837, OLMALINC and AL669970.3 were mainly expressed in the high-risk groups, and may reduce the overall survival.

Previous research has identifed ELFN1-AS1 was overexpressed in tumors and indicated that it might play an essential role in carcinogenesis (29). Importantly, Down-regulation of ELFN1-AS1 could inhibit the proliferation and migration of tumor cells in esophageal cancer and colorectal cancer. ELFN1-AS1 promoted tumor cell proliferation and metastasis by acting as a sponge of miR-183-3p to upregulate GFPT1 in esophageal cancer (30). ELFN1-AS1 could promote proliferation, metastasis and exert anti-apoptosis effect by up-regulating TRIM44 by sponging miR-4644 in colorectal cancer (31). However, till now the specific function of the lncRNA remains unknown in osteosarcoma. In our study, we found that ELFN1-AS1 was negatively related to plasma cells. Plasma cells have been shown to be positively associated with patient prognosis in colon cancer, non-small cell lung cancer and triple-negative breast cancer (32–34). We speculate that ELFN1-AS1 may influence the development of osteosarcoma by affecting the proliferation and function of plasma cells, and the exact mechanism remains to be further investigated. OLMALINC was reported to overexpress in the white matter of the human brain, which played a role in maintaining the maturation of oligodendrocytes (35). In our study, we found that OLMALINC was positively related to activated mast cells and CD4^+^ naive T cells, and negatively related to memory B cells. Mast cells have been observed to increase in tumor and peritumor tissues (36). However, Mast cells have different roles in different types of tumors. Mast cells were associated with promoting tumorigenesis in bladder cancer, colorectal cancer, lung cancer and hepatocellular cancer (37–40). Mast cells have antitumor activities in breast cancer, diffuse large B-cell lymphoma and ovarian cancer (41–43). Naive T cells were reported to express functional CXCL8 and promote tumorigenesis (44). Blocking naive CD4^+^ T cell recruitment into tumors reversed immunosuppression in breast cancer, which may be an attractive strategy for antitumor treatment (45). Memory B cells were associated with a good prognosis in gastric cancer and non-small cell lung cancer (46, 47). These studies were consistent with our results. OLMALINC may affect osteosarcoma development by inhibiting memory B cell proliferation and function, recruiting mast cells, and naive CD4^+^ T cells.

While, there was little research studied on the role of LINC00837, AL669970.3, AC005332.4 and AC023157.3. In our study, we found that LINC00837 was positively related to resting dendritic cells. Dendritic cells are the most important antigen-presenting cells in the immune system, playing a key role in regulating immunity. However, resting dendritic cells can induce immune tolerance through T cell deletion and induction of regulatory T cells (48). AL669970.3 was positively related to activated T CD4^+^ memory cells. But this is contrary to our common perception that activated T CD4^+^ memory cells can positively modulate immune function and inhibit tumor growth (49). It warrants further investigation. AC023157.3 was positively related to monocytes and M2 macrophages, while negatively related to memory B cells and M0 macrophages. Using a three-dimensional vascularized microfluidic model, Boussommier-Calleja et al. demonstrated that monocytes were able to directly reduce cancer cell extravasation, but that such an effect was lost when monocytes were converted to macrophages (50). Many studies have suggested that M2 polarized tumor-associated-macrophages (TAM) are associated with tumor growth, invasion, and metastasis (51, 52). However, Anne Gomez-Brouchet et al. found that the presence of CD163-positive M2-polarized macrophages was critical to inhibit OS progression (53). This suggests that M2-polarized macrophages may be influenced by other factors to exert distinctly different pro- or anti-tumor effects. Zhang et al. indicated that the level of polarization of M0 to M1 or M2 macrophages may be an important factor (54). It was also reported that the balance between M1 and M2 macrophage would affect the PD-1/PDL-1, which is an important immune regulatory system (55). In addition, we found that among these six RNAs, AC023157.3 was highly expressed in patients without metastasis. We speculate that AC023157.3 may inhibit tumor growth and metastasis by promoting the proliferation and function of monocytes and by regulating the level of polarization of M0 to M1 or M2 macrophages. The specific mechanism deserves further investigation. Combined with the above findings, we found that all the six RNAs in the signature had some relationship with tumorigenesis or the immune system, which could provide a theoretical basis for immunotherapy of osteosarcoma.

We analyzed the enrichment of immune-related pathways in high- and low-risk groups by GSEA analysis, and found that the immune-related pathways were mainly enriched in the low-risk group, suggesting that the development of osteosarcoma may be highly associated with a lack of immune response. ESTIMATE analysis showed that the low-risk group contained more immune cells with a higher immune score and ESTIMATE score, indicating the lower tumor purity. The risk score was also negatively correlated with immune score, stromal score and ESTIMATE score. The results indicated that risk scores were positively correlated with tumor purity. There were studies reported that a lower tumor purity and more immune cell components mean a poor prognosis (19, 54). However, it was different in osteosarcoma that patients with increased microenvironmental immune cell infiltration had a better prognosis (54), which was consistent with our results. It was reported that the main components of the immune environment in osteosarcoma are T-lymphocytes and macrophages (55). CIBERSORT analysis in our study showed similar results. There were more mature lymphocytes, including CD8^+^ T cells (p=0.041) and CD4^+^ activated memory T cells, enriched in the low-risk group. Meanwhile, CD4^+^ naive T cells were enriched in the high-risk group. The risk scores were also negatively correlated to CD8^+^ T cells, while positively correlated to CD4^+^ naive T cells. These results suggested immature immune function in the high-risk group might be an important factor for the development of osteosarcoma. Therefore, targeting the immune system and changing the composition and proportion of immune cells in the tumor microenvironment is a promising treatment option for osteosarcoma.

However, our study has limitations. First, the sample sizes of osteosarcoma are small. More data is needed to verify the accuracy of the signature. Second, because the microarray data for osteosarcoma are relatively scarce and the platforms used are relatively old, which are not sensitive enough for lncRNA, we did not find other external cohorts with survival information to validate the signature. Third, we need further experiments, such as immunohistochemical analysis, PCR, or western blot, to support these findings. Despite these limitations, we constructed a six lncRNAs signature with a good prognostic value in osteosarcoma. These lncRNAs may play essential roles in the progression of osteosarcoma. They deserve further study.

## Conclusion

In conclusion, we identified an immune-related lncRNAs for osteosarcoma, which possess good performance in the prediction of survival, metastasis and immune microenvironment. Our study not only has great significance in predicting the prognosis but also has potential to guide future immunotherapy in osteosarcoma.

## Data Availability Statement

Publicly available datasets were analyzed in this study. This data can be found here: https://portal.gdc.cancer.gov/.

## Author Contributions

HC and YH performed the conception and design of this manuscript. HZ provided useful suggestions in methodology. HZ and HX performed data analysis and prepared the figures. YH drafted the manuscript. HC and HY revised the manuscript. All authors read and approved the final manuscript.

## Conflict of Interest

The authors declare that the research was conducted in the absence of any commercial or financial relationships that could be construed as a potential conflict of interest.

## Publisher’s Note

All claims expressed in this article are solely those of the authors and do not necessarily represent those of their affiliated organizations, or those of the publisher, the editors and the reviewers. Any product that may be evaluated in this article, or claim that may be made by its manufacturer, is not guaranteed or endorsed by the publisher.

## Abbreviations

LncRNA, Long non-coding RNA; TCGA, The Cancer Genome Atlas; ROC, Operating characteristic curve; PCA, Principal component analysis; GSEA, Gene set enrichment analysis; AUC, Area under curve; LASSO, Least absolute shrinkage and selection operator; K-M, Kaplan–Meier.
